# Cholangioscopic findings in complicated liver hydatid disease

**DOI:** 10.1055/a-2387-4169

**Published:** 2024-09-04

**Authors:** Joana Revés, Bénédicte Delire, Alice Dongier, Pierre Deprez

**Affiliations:** 1 467035 Gastroenterology, Hospital Beatriz Ângelo, Loures, Portugal; 2 Hepatogastroenterology, Cliniques Universitaires Saint-Luc, Université Catholique de Louvain, Brussels, Belgium

A 57-year-old man was incidentally diagnosed with a liver hydatid cyst during a magnetic resonance imaging (MRI) scan. Follow-up examination revealed cyst enlargement to 10 cm, with multiloculation and slight dilation of the right anterior intrahepatic bile duct. The patient underwent surgery after he had received two months of albendazole therapy. The surgical protocol involved nonanatomic hepatectomy of the cyst following hypertonic saline injection and pericystectomy. Intraoperatively, a biliary fistula was discovered.

The patient experienced multiple episodes of cholangitis two months after his surgery. Cross-sectional imaging revealed a biliary fistula with biloma and suspected disease recurrence, prompting endoscopic retrograde cholangiopancreatography (ERCP) with sphincterotomy and stent insertion for bilateral hilar strictures. For better characterization of the strictures, a subsequent cholangioscopy (SpyGlass DS; Boston Scientific, Belgium) was performed ( Video 1 ), which revealed a hilar cavity and severe biliary wall ulceration ( Fig. 1 ) running from the common hepatic duct to the right and left hepatic ducts, with yellowish rounded structures within the ducts that were suggestive of daughter vesicles. An endoscopic ultrasound (EUS) further assessed the cavity, revealing a dilated remnant of the cystic duct with hypoechogenic rounded vesicles inside that were again suggestive of daughter vesicles. Following the insertion of multiple stents, the patient improved, with no further episodes of cholangitis, although his cholestasis persisted.

**Fig. 1:**
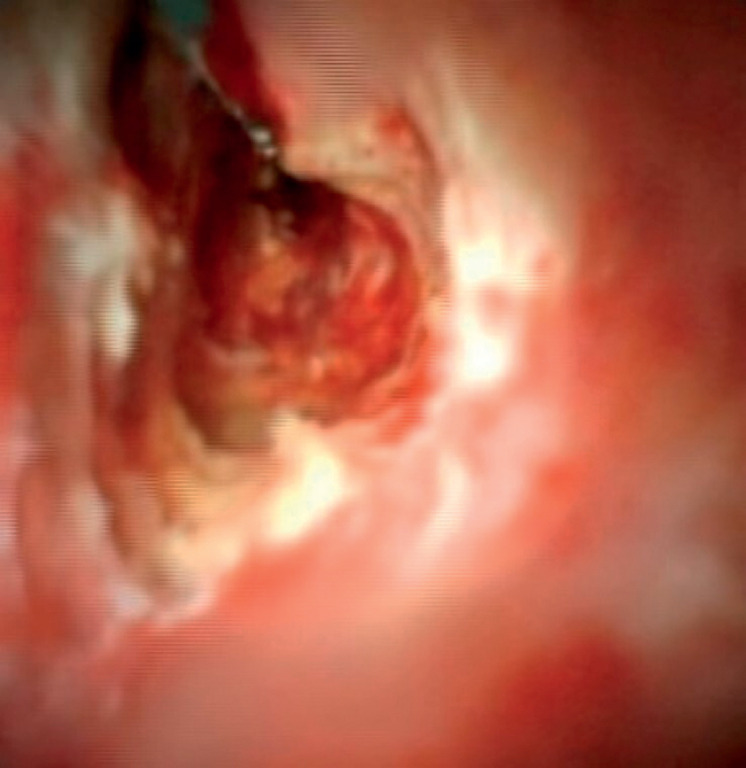
Cholangioscopic image showing severe biliary wall ulceration.

Cholangioscopic and endoscopic ultrasound findings of possible caustic sclerosing cholangitis caused by scolicidal agents and daughter vesicles of a complicated liver hydatid cyst.Video 1

Hepatic alveolar echinococcosis, which is endemic in many regions, may result in a cystobiliary communication in up to 42% of patients, complicating post-surgical outcomes when it is undiagnosed. Cystobiliary communication can be associated with obstructive jaundice, cholangitis, pancreatitis, cholecystitis, or anaphylactic shock due to the migration of daughter vesicles or cyst membrane 1 . ERCP plays a crucial role in managing these complications, both pre- and postoperatively 2 3 . While scolicidal agents are effective, they may lead to caustic sclerosing cholangitis, particularly in the presence of a cystobiliary communication. Caustic sclerosing cholangitis progresses rapidly and may necessitate liver transplantation 4 .

Our case highlights the unusual cholangioscopic findings of biliary damage due to caustic sclerosing cholangitis and the presence of daughter vesicles, representing a unique contribution to the literature.

Endoscopy_UCTN_Code_TTT_1AR_2AB

## Notes

Endoscopy E-Videos https://eref.thieme.de/e-videos
*E-Videos* is an open access online section of the journal *Endoscopy* , reporting on interesting cases and new techniques in gastroenterological endoscopy. All papers include a high-quality video and are published with a Creative Commons CC-BY license. Endoscopy E-Videos qualify for HINARI discounts and waivers and eligibility is automatically checked during the submission process. We grant 100% waivers to articles whose corresponding authors are based in Group A countries and 50% waivers to those who are based in Group B countries as classified by Research4Life (see: https://www.research4life.org/access/eligibility/ ). This section has its own submission website at https://mc.manuscriptcentral.com/e-videos .
